# Promising Effects of Casearins in Tumor-Bearing Mice and Antinociceptive Action against Oncologic Pain: Molecular Docking and In Vivo Findings

**DOI:** 10.3390/ph17050633

**Published:** 2024-05-14

**Authors:** Jurandy do Nascimento Silva, José Ivo Araújo Beserra Filho, Boris Timah Acha, Fernanda Regina de Castro Almeida, Emanuelle Karine Frota Batista, Valdenizia Rodrigues Silva, Larissa Mendes Bomfim, Milena Botelho Pereira Soares, Daniel Pereira Bezerra, André Gonzaga dos Santos, Francisco das Chagas Pereira de Andrade, Anderson Nogueira Mendes, Daniel Dias Rufino Arcanjo, Paulo Michel Pinheiro Ferreira

**Affiliations:** 1 Laboratory of Experimental Cancerology (LabCancer), Department of Biophysics and Physiology, Federal University of Piauí, Teresina 64049-550, Brazil; jurandy@ufpi.edu.br (J.d.N.S.); ftjoseivo@gmail.com (J.I.A.B.F.); 2 Department of Chemistry, Federal University of Piauí, Teresina 64049-550, Brazil; 3 Laboratory of Functional and Molecular Studies in Physiopharmacology (Lafmol), Department of Biophysics and Physiology, Federal University of Piauí, Teresina 64049-550, Brazil; timah.boris@yahoo.com; 4 Laboratory of Pain Pharmacology, Department of Biochemistry and Pharmacology, Federal University of Piauí, Teresina 64049-550, Brazil; ferecal@ufpi.edu.br; 5 Animals Veterinary Hospital, Teresina 64048-180, Brazil; emanuellefrota@yahoo.com.br; 6 Laboratory of Tissue Engineering and Immunopharmacology, Oswaldo Cruz Foundation, Salvador 40296-710, Brazil; valdeniziar@gmail.com (V.R.S.); larissambomfim@gmail.com (L.M.B.); milenabpsoares@gmail.com (M.B.P.S.); danielpbezerra@gmail.com (D.P.B.); 7 Laboratory of Pharmacognosy, Faculty of Pharmaceutical Sciences, State University Júlio de Mesquita Filho, Araraquara 14800-700, Brazil; andre.gonzaga@unesp.br; 8 Laboratory of Innovation in Science and Technology (Lacitec), Department of Biophysics and Physiology, Federal University of Piauí, Teresina 64049-550, Brazil; profandradefc@gmail.com (F.d.C.P.d.A.); anderson.mendes@ufpi.edu.br (A.N.M.)

**Keywords:** anticancer action, clerodane diterpenes, colorectal carcinoma, glutamate receptors, sarcoma 180

## Abstract

Safer analgesic drugs remain a hard challenge because of cardiovascular and/or gastrointestinal toxicity, mainly. So, this study evaluated in vivo the antiproliferative actions of a fraction with casearins (FC) from *Casearia sylvestris* leaves against human colorectal carcinomas and antihyperalgesic effects on inflammatory- or opiate-based pain relief and oncologic pain in Sarcoma 180 (S180)-bearing mice. Moreover, docking investigations evaluated the binding among Casearin X and NMDA(N-methyl-D-aspartate)-type glutamate receptors. HCT-116 colorectal carcinoma-xenografted mice were treated with FC for 15 days. Antinociceptive assays included chemically induced algesia and investigated mechanisms by pharmacological blockade. Intraplantar region S180-bearing animals received a single dose of FC and were examined for mechanical allodynia and behavior alterations. AutoDock Vina determined molecular interactions among Cas X and NMDA receptor subunits. FC reduced tumor growth at i.p. (5 and 10 mg/kg) and oral (25 mg/kg/day) doses (31.12–39.27%). FC reduced abdominal pain, as confirmed by formalin and glutamate protocols, whose antinociception activity was blocked by naloxone and L-NAME (neurogenic phase) and naloxone, atropine, and flumazenil (inflammatory phase). Meanwhile, glibenclamide potentiated the FC analgesic effects. FC increased the paw withdrawal threshold without producing changes in exploratory parameters or motor coordination. Cas X generated a more stable complex with active sites of the NMDA receptor GluN2B subunits. FC is a promising antitumor agent against colorectal carcinomas, has peripheral analgesic effects by desensitizing secondary afferent neurons, and inhibits glutamate release from presynaptic neurons and/or their action on cognate receptors. These findings emphasize the use of clerodane diterpenes against cancer-related pain conditions.

## 1. Introduction

Cancer is a multifactorial disease characterized by the uncontrolled division of cells involving the accumulation of inherited or somatic mutations in oncogenes, gene suppressors, and DNA repair genes and relying on the interaction with environmental factors, including diet, infections, and work exposure [1,2]. The progress of the carcinogenic process promotes the accumulation of chromosomal errors, metastasis, and loss of normal organic functions [3].

The symptoms of cancer include pain, infections, cough, dyspnea, fatigue, haematuria, haemoptysis, hoarseness, and cachexia [4]. About 20 to 50% of cancer patients have pain whose intensity depends on the location, type of cancer, and treatment. The occurrence of this symptom increases according to the progression of the disease, reaching up to 90% in terminally ill patients [5,6], and the painful condition is associated with behavioral comorbidities such as anxiety and depression [7]. Moreover, pain is also a side effect of chemotherapeutics and radiotherapy, as are peripheral neuropathy, mouth sores (stomatitis or mucositis), and radiation dermatitis and mucositis [8].

Pharmacological management of cancer pain is associated with the prescription of non-steroidal anti-inflammatory drugs (e.g., acetaminophen, celecoxib, diclofenac, ibuprofen, and ketoprofen) to relieve mild pain. However, the chronic use of these drugs is associated with cardiovascular and gastrointestinal toxicity, nephrotoxicity, hepatotoxicity, and hematologic effects [9]. Meanwhile, opioids are commonly used to relieve moderate to severe pain, mainly morphine, codeine, fentanyl, oxycodone, and tramadol. Opioids are more effective but cause nausea, drowsiness, dry mouth, and constipation. They can also cause physical dependence (tolerance), and up to 8% of cancer patients may be addicted to opioids [10,11]. Then, effective and safer new analgesic drugs remain a hard challenge for biologists, pharmacologists, and oncologists.

In this context, natural products have been used as a source of novel anticancer [12,13,14] and antinociceptive [15] agents. Among them, *Casearia* (Salicaceae) comprises about 180 species distributed in sub-tropical and tropical regions [16] and has promising anticancer effects [17,18,19]. In Brazil, *Casearia sylvestris* Sw. is the most known species and has shown antifungal [20], anxiolytic [21], antibacterial [22], antioxidant [23,24], antileishmanial [25], anti-inflammatory [26,27], and antimicrobial [24,28] activities.

Most pharmacological effects of *C. sylvestris* are attributed to the secondary metabolites named clerodane diterpenes, including casearins, casearvestrins, caseazins, and caseargrewiins from leaves [18,29,30,31,32,33]. Our research group has worked for about 20 years with this plant and has shown casearins identified by bioguided assays from *C. sylvestris* leaves to have strong in vitro antiproliferative activity on different tumor cell lines [18,31,34]. We also displayed the fraction from *C. sylvestris* leaves that showed in vivo tumor inhibition rates ranging from 33 to 67% for human carcinomas and glioblastomas and from 35 to 90% for Sarcoma 180 murine (S180) cells [19]. Then, considering the mixture of clerodane diterpenes represents the most common approach in ethnopharmacological uses by the Brazilian population and its promising phytochemical properties for the development of phytotherapy drugs, the present study evaluated in vivo the antiproliferative actions of a fraction with casearins (FC) from *C. sylvestris* leaves against human colon carcinomas and the antihyperalgesic effects on inflammatory- or opiate-based pain relief and oncologic pain in Sarcoma 180-bearing mice. Moreover, molecular docking investigations evaluated the binding among Casearin X and NMDA-type glutamate receptors.

## 2. Results

### 2.1. Fraction with Casearins Has Antitumor Activity in a Xenographic Model of Human Colon Carcinoma without Systemic Side Effects

No statistical differences in the relative wet weight of organs were noted when compared to the negative controls (i.p. and oral), though 2 deaths occurred after 5-FU exposure (Table 1). No changes were detected in weight gain and hematological parameters (Table 2) in FC-treated animals, but mice receiving 5-FU presented leukopenia. Meanwhile, FC reduced tumor growth at both i.p. doses (5 and 10 mg/kg: 0.69 ± 0.03 and 0.74 ± 0.05 g) and at the highest oral dose (25 mg/kg/day: 0.70 ± 0.06 g) when compared to the negative group (1.08 ± 0.07 and 1.06 ± 0.04 g, respectively) (*p* < 0.05).

### 2.2. Histopathological Analysis

**Figure 1:**
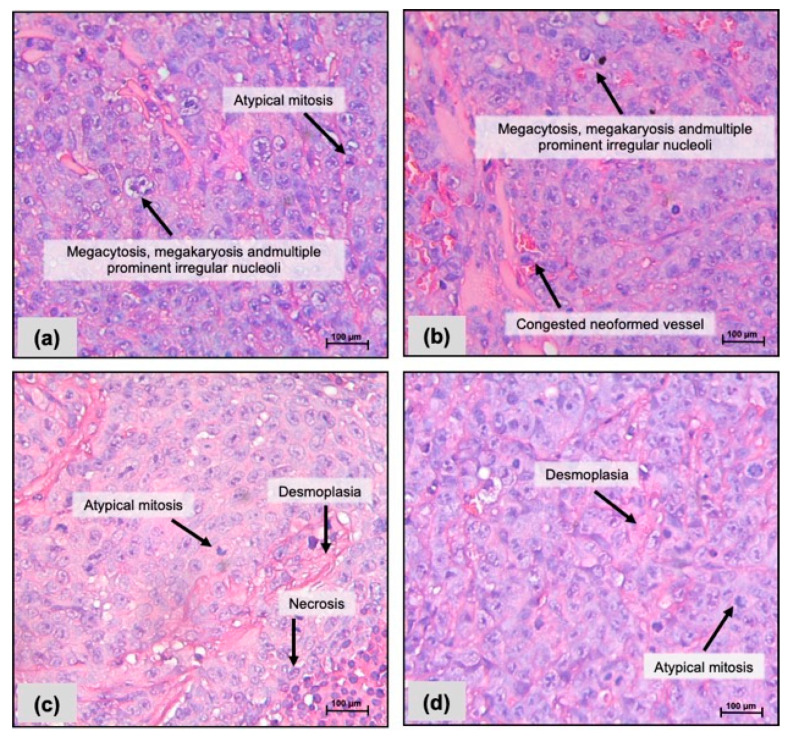
Photomicrographs of human colon carcinoma cells (HCT-116) inoculated in CB-17 SCID mice after 15 days of treatment with a fraction with casearins (FC). (**a**,**b**) Negative controls were treated with the vehicle used to dilute the substance (DMSO 5% i.p. and oral gavage, respectively). (**c**) FC 10 mg/kg i.p.; (**d**) FC 10 mg/kg oral. Hematoxylin–eosin staining. Scale = 100 µm.

Morphological analysis performed on human colon carcinoma tumor (HCT-116 cells) revealed higher levels of anaplasia, megacytosis and megakaryosis, multiple, prominent, and irregular nucleoli, fibrous tissue (desmoplasia), and atypical mitoses in all groups (Figure 1a–d). Congested new-formed vessels were more common in the negative control group treated orally, and necrotic cell death occurred in FC-treated animals at 10 mg/kg i.p. (Figure 1c).

**Figure 2:**
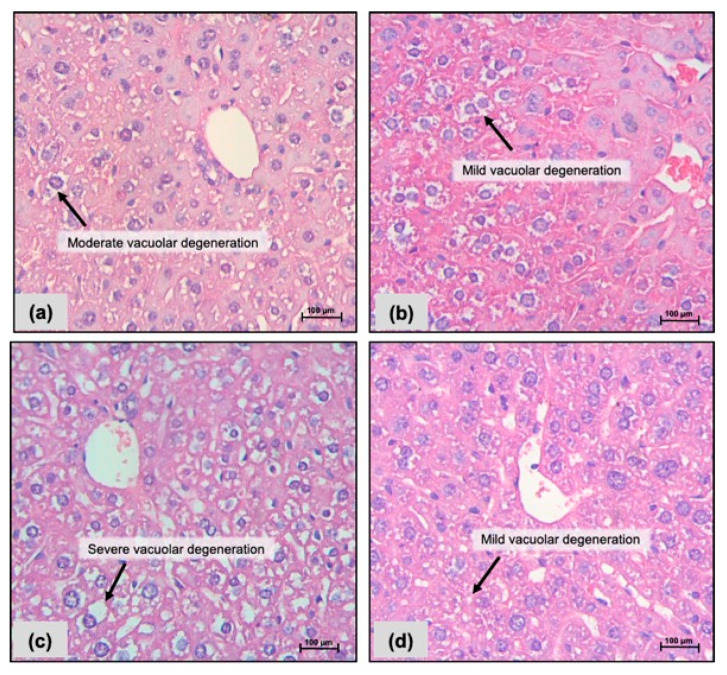
Photomicrographs of livers from CB-17 SCID mice after 15 days of treatment with a fraction with casearins (FC). (**a**,**b**) Negative controls were treated with the vehicle used to dilute the substance (DMSO 5% i.p. and oral gavage, respectively). (**c**) FC 10 mg/kg i.p.; (**d**) FC 10 mg/kg oral. Hematoxylin–eosin staining. Scale = 100 µm.

**Figure 3:**
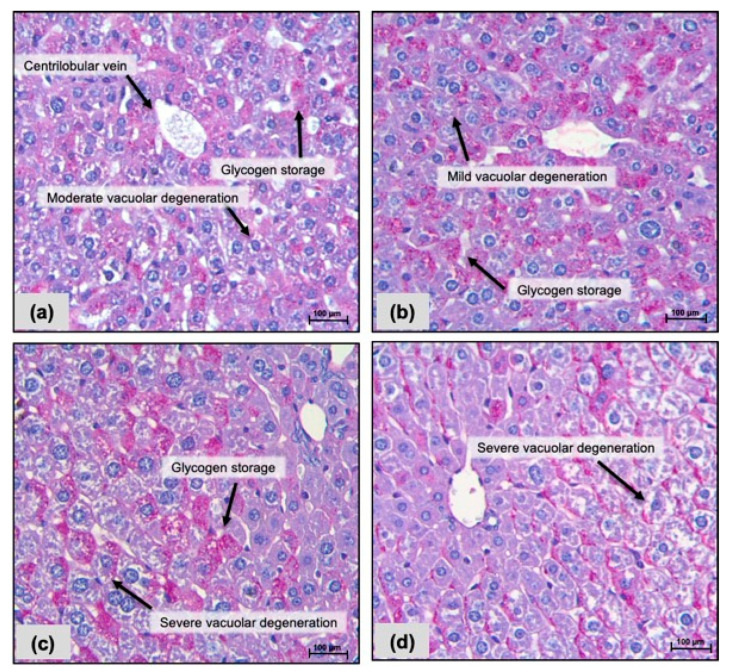
Photomicrographs of livers from CB-17 SCID mice after 15 days of treatment with a fraction with casearins (FC). (**a**,**b**) Negative controls were treated with the vehicle used to dilute the substance (DMSO 5% i.p. and oral gavage, respectively). (**c**) FC 10 mg/kg i.p.; (**d**) FC 10 mg/kg oral. Periodic acid–Schiff staining. Scale = 100 µm.

**Figure 4:**
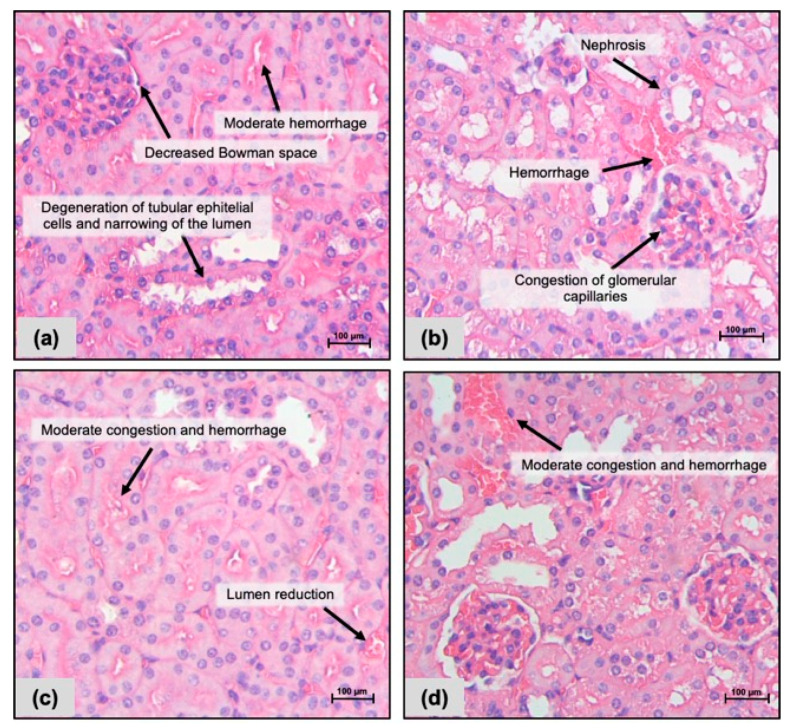
Photomicrographs of kidneys from CB-17 SCID mice after 15 days of treatment with a fraction with casearins (FC). (**a**,**b**) Negative controls were treated with the vehicle used to dilute the substance (DMSO 5% i.p. and oral gavage, respectively). (**c**) FC 10 mg/kg i.p.; (**d**) FC 10 mg/kg oral. Hematoxylin–eosin staining. Scale = 100 µm.

Livers revealed the presence of vacuolar degeneration in all groups, but such alteration was more observed in the group receiving FC 10 mg/kg i.p. (Figure 2c). Additionally, liver sections stained with periodic acid–Schiff (PAS) showed more common glycogen deposits and vacuolar degeneration in those groups exposed to FC 10 mg/kg/day i.p. and oral (Figure 3c,d). Hemorrhage was observed within kidneys in all groups, but cell congestion was observed in FC-treated mice (i.p. and oral: Figure 4c,d) only; a decrease in the lumen of renal tubules was found after exposure to FC i.p. (Figure 4c).

### 2.3. Antinociceptive Action against Chemically Induced Pain

**Figure 5:**
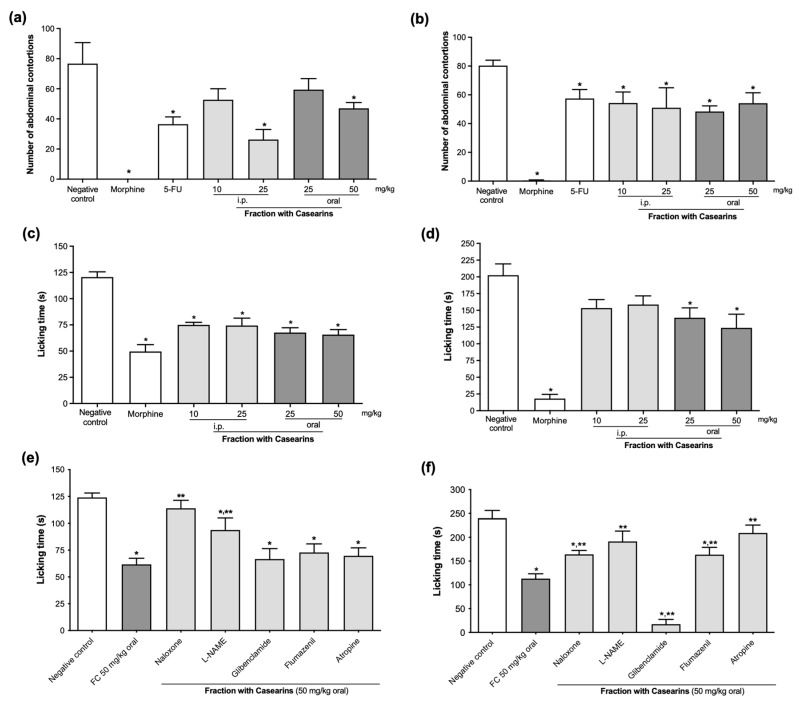
Evaluation of the antinociceptive effects of the fraction with casearins (FC) obtained from *Casearia sylvestris* leaves. (**a**) Acetic acid-induced abdominal writhing test after a single dose of FC. (**b**) Acetic acid-induced abdominal writhing test after 7 days of treatment with FC. (**c**) Neurogenic phase of the formalin-induced pain after a single dose of FC. (**d**) Inflammatory phase of the formalin-induced pain after a single dose of FC. (**e**) Formalin-induced pain in the neurogenic phase after i.p. treatment with blockers (naloxone 5 m/kg, L-NAME 20 mg/kg, glibenclamide 10 mg/kg, flumazenil 2 mg/kg, and atropine 1 mg/kg) followed by FC 50 mg/kg oral. (**f**) Formalin-induced pain in the inflammatory phase after i.p. treatment with blockers followed by FC 50 mg/kg oral. Negative control was treated with the vehicle used to dilute the drug (DMSO 5%). Morphine (5 mg/kg i.p.) and 5-FU (25 mg/kg i.p.) were used as positive controls. Values are means ± S.E.M., *n* = 7 animals/group. * *p* < 0.05 compared with the negative control by ANOVA followed by the Newman–Keuls test; ** *p* < 0.05 compared to the FC group by ANOVA followed by the Newman–Keuls test.

Firstly, the antinociceptive effects were evaluated in Swiss mice acutely treated with a single dose of FC. Both highest doses (25 mg/kg i.p. and 50 mg/kg oral) reduced episodes of retraction of the abdomen and stretching of hind limbs induced by acetic acid (26.3 ± 6.6 and 47.0 ± 3.9, respectively) when compared to the negative control (76.7 ± 14.0; *p* < 0.05). Such a reduction was also observed in 5-FU-treated animals (36.5 ± 4.9 s) and morphine (they did not present writhing) (Figure 5a). In similar experimental conditions, all subacute intraperitoneal and oral doses of FC for 7 days declined contortions when compared to the negative control (*p* < 0.05, Figure 5b).

All FC doses (10 and 25 mg/kg i.p.; 25 and 50 mg/kg oral) reduced the paw licking time (75.0 ± 2.5 s; 74.4 ± 7.0 s; 67.7 ± 4.6 s; and 65.7 ± 4.8 s, respectively) in the neurogenic phase of the formalin test (0–5 min) when compared to the negative control (120.6 ± 4.9 s) as well as morphine (49.7 ± 6.6 s; *p* < 0.05) (Figure 5c). In the second inflammatory phase (15–30 min), only FC oral (25 and 50 mg/kg) exhibited antinociceptive efficacy (139.0 ± 14.7 and 123.0 ± 20.5 s) in comparison with the negative control group (202.5 ± 16.86 s; *p* < 0.05) (Figure 5d).

Pre-treatment with the opioid receptor antagonist naloxone and L-arginine analogue nitric oxide synthase inhibitor L-NAME before a single dose of FC 50 mg/kg oral reversed the antinociceptive effects demonstrated by the fraction (113.9 ± 7.5 s and 93.9 ± 10.9 s) during the formalin test first phase when compared to the group receiving FC (61.8 ± 5.7 s; *p* < 0.05). On the other hand, glibenclamide, flumazenil, and atropine did not reverse the reduction in licking paw time induced by FC (Figure 5e) during the neuronal-dependent stage.

In the inflammatory phase, naloxone (164.1 ± 8.3 s), L-NAME (191.3 ± 21.9 s), atropine (209.3 ± 16.59 s), and the benzodiazepine antagonist flumazenil (163.5 ± 15.4 s) reversed the antinociceptive action of FC 50 mg/kg oral (113.1 ± 10.3 s, Figure 5f). Interestingly, the association of glibenclamide with FC potentiated the analgesic results of FC (17.3 ± 9.9 s) if compared to the FC group alone (*p* < 0.05).

### 2.4. Fraction with Casearins Reduces Oncologic Pain in S180-Transplanted Mice

**Figure 6:**
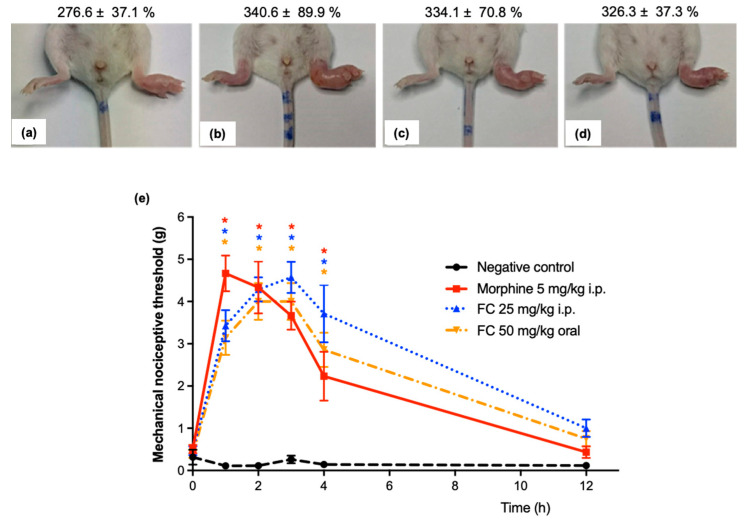
Evaluation of direct nociception in the right hind paw of Sarcoma180-bearing mice following acute treatment with a fraction with casearins (FC) extracted from Casearia sylvestris leaves on the 14th day after the tumor inoculation. (**a**–**d**) Paw growth (%) measured by plethysmometer after comparison of paw volume to its counterpart on the 14th day after tumor inoculation. (**e**) Each point represents the mechanical sensitivity score analyzed by the Von Frey filament technique (from 0.07 to 10 g) (*n* = 7 animals/group). Negative control (vehicle group) was received at DMSO 5%. * *p* < 0.05 compared with the negative control by ANOVA followed by the Newman–Keuls test.

The size of each paw was compared by plethysmometer with its paw volume at the beginning of the study (before tumor induction) to monitor tumor growth and ensure there were no statistical differences (*p* > 0.05) between groups for acute treatments. Indeed, 14 days following intraplantar S180 transplantation, tumors were well established macroscopically (Figure 6a) and showed similar evolution (Figure 6a–d). So, mice were divided into four groups: negative control (276.6 ± 37.1% of S180-induced paw growth), morphine (340.6 ± 89.9%), FC 25 mg/kg i.p. (334.1 ± 70.8%), and FC 50 mg/kg oral (326 ± 37.7%) (*p* > 0.05).

Then, the antiallodynic effects of FC were analyzed using the Von Frey test. Initially, S180-induced hypernociception was observed when the mechanical sensitivity threshold was compared between the negative group and morphine-treated animals (*p* < 0.05). Next, FC 25 mg/kg i.p. and 50 mg/kg orally increased the paw withdrawal threshold until 240 min after treatment in comparison with the negative group (*p* < 0.05). However, 12 h later, no differences were noted among groups (*p* > 0.05) (Figure 6e).

**Figure 7:**
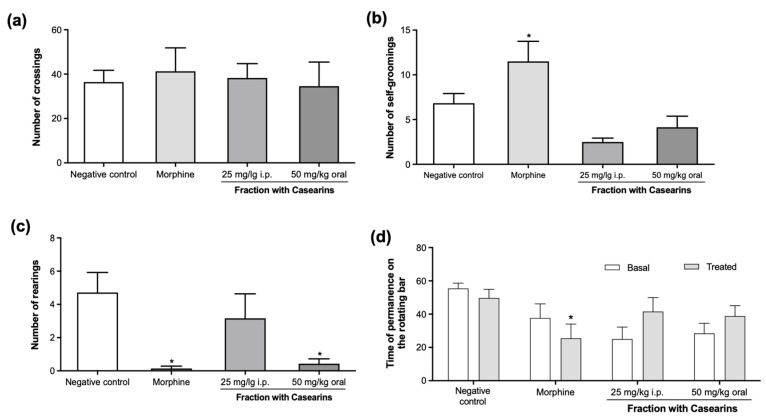
Behavioral examinations in Sarcoma180-bearing mice following acute treatment with a fraction with casearins (FC) extracted from *Casearia sylvestris* leaves on the 14th day after the tumor inoculation. The analysis included exploratory follow-ups by the open field task (**a**–**c**) and locomotor activity by the rotarod apparatus (**d**). Negative control (vehicle group) was received at DMSO 5%. * *p* < 0.05 compared with the negative control by ANOVA followed by the Newman–Keuls test.

Figure 7a shows results on exploratory and locomotor activity using open field tests. Although no differences were found in the number of crossings (*p* > 0.05), animals treated with morphine (5 mg/kg i.p.) and FC 50 mg/kg oral presented a decrease in the number of rearings (0.1 ± 0.1 and 0.4 ± 0.2) when compared to the negative control (*p* < 0.05) (Figure 7c). On the other hand, FC did not modify motor coordination parameters examined in the rotarod apparatus (Figure 7d, *p* > 0.05).

### 2.5. Casearin X Binds to NMDA-Type Glutamate Receptors

**Figure 8:**
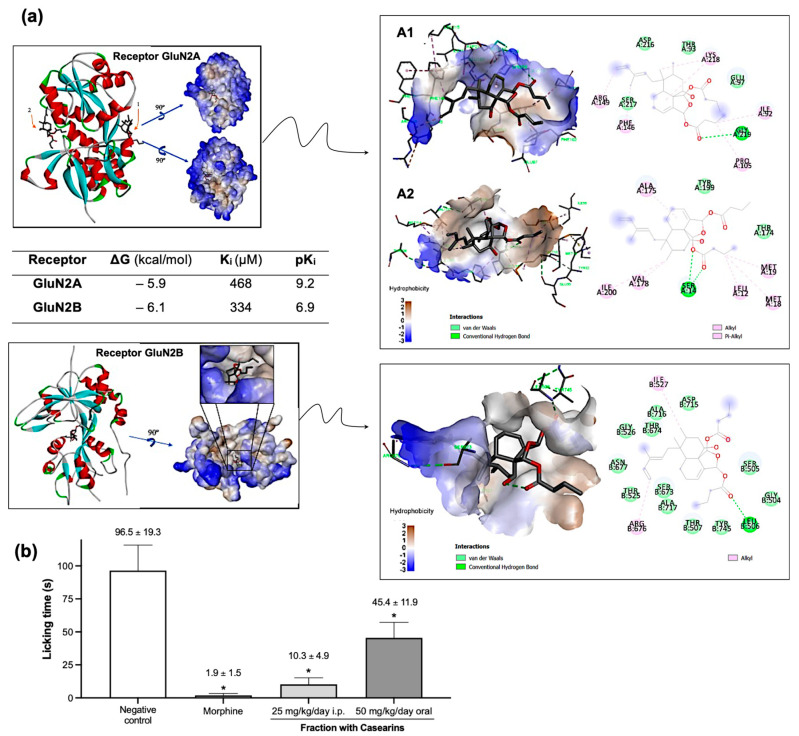
Molecular docking and pharmacophoric map showing the ability of Casearin X, the main molecule in the fraction with casearins from *Casearia sylvestris* leaves, to form a complex with NMDA receptors (GluN2A and GluN2B). (**a**) Values of Gibbs free energy (∆G°), inhibition constant (Ki), and pKi for complexes among Casearin X and ionotropic NDMA glutamatergic receptors. Structures that are thermodynamically more stable after analysis of molecular docking between Casearin X and each receptor are also displayed. (**b**) Evaluation of the antinociceptive effects of the fraction with casearins (FC) obtained from *Casearia sylvestris* leaves on glutamate-induced pain after a single dose. Negative control was treated with the vehicle used to dilute the drug (DMSO 5%). Morphine (5 mg/kg i.p.) was used as a positive control. Values are means ± S.E.M., *n* = 8 animals/group. * *p* < 0.05 compared with the negative control by ANOVA followed by the Newman–Keuls test.

Once again, both doses of FC (25 mg/kg i.p. and 50 mg/kg oral) reduced the paw licking time induced by glutamate (10.3 ± 4.9 and 45.4 ± 11.9 s) when compared to the negative control (96.5 ± 19.3 s, respectively) (Figure 8b). These in vivo findings were supported by molecular docking, which indicates the ability of Cas X to form a complex with both GluN2A and GluN2B protein structures. However, Cas X displayed better stability after interacting with active sites of GluN2B receptors, as indicated by binding energy (ΔG) values and enzyme activity inhibition constants (Ki) (Figure 8a). The interactions of Cas X with the ligand-binding extracellular domain site of GluN2B occur through hydrogen bonds with Leu-506, alkyl interactions with residues of Ile-527 and Arg-676, and by van der Waals forces with Gly-504, Ser-505, Thr-507, Thr-525, Gly-526, Ser-673, Thr-674, Asn-677, Asp-715, Ala-716, and Tyr-745.

## 3. Discussion

The identification of pharmacologically active compounds from plants and other natural sources and the determination of mechanism(s) of action are the main challenges for the development of pharmaceutical chemistry and breaking down pharmacokinetic barriers [14,35,36].

### 3.1. Anticancer Action and Toxicological Issues

With regard to the species *C. sylvestris*, phytochemical studies have isolated different diterpenes with remarkable in vitro and in vivo activity upon different human and murine cancers, such as Ehrlich, Lewis, S180, leukemias (HL-60, CEM, K-562), colon (HCT, HCT-8, HCT-116), breast (MDA/MB-231, Hs578-T, MX-1, MCF-7), melanoma (MDA/MB-435, A2058, B-16/F10, B16F10-Nex2), prostate (PC-3, DU-145), ovary (A-2780), lung (LX-1, A-549), cervical carcinoma (HeLa), and glioblastoma (SF-295) [18,19,29,37,38,39,40,41,42,43]. Additionally, our research group has shown the ethyl acetate fraction from *C. sylvestris* leaves as a source of cytotoxic clerodane-type diterpenes, mainly Casearin X and Caseagrevin F [19,34].

So, initially, we assessed the anticancer action of FC in human colon carcinoma-xenografted mice. This fraction reduced tumor mass from 31.12 to 39.27%, which was corroborated by the expansion of necrotic areas in tumors from FC-treated mice. Such outcomes confirmed prior results in nude transplanted mice with hollow fibers of polyvinylidene fluoride (Hollow Fiber Assay, HFA) bearing HCT-116 cancer cells, whose findings indicated tumor reductions from 48.5 to 70.3% [19]. It also showed ethanolic extract [37] and FC [19] from *C. sylvestris* leaves at doses ranging from 10 to 100 mg/kg/day have promising in vivo antiproliferative activity in Sarcoma 180-transplanted mice (tumor reductions from 87% to 98% for ethanolic extract and from 35.8 to 90% for FC). Similar anticancer results were found with ethanolic extract [44], crude aqueous ethanolic extract, and chloroform fraction [45] against Ehrlich tumors and with gallic acid-derived compounds isolated from *C. sylvestris* leaves on Ehrlich and Lewis lung cancer ascite cells [40].

Some studies have demonstrated a direct correlation between activity in HFA and xenographic models, indicating an increased probability for successful outcomes in Phase II clinical trials for cytotoxic drugs. A substance is considered active and demonstrates anticancer efficacy in a particular type of cancer if it reduces cell growth by 50% or more [46].

Interestingly, the FC anticancer action on colon carcinomas was not associated with signs/symptoms of systemic toxicity, inasmuch as changes in body weight, the relative weight of organs, or hematological or histological records were not observed. Previously, we showed that FC 5 and/or 10 mg/kg/day i.p. for 30 days caused anemia, lymphocytopenia, neutrophilia, hypoalbuminemia, hepatomegaly, an increase in ALT and diminished albumin, alkaline phosphatase, glucose levels, and relative weight of the heart. Casearin X-treated mice presented lymphocytopenia and neutrophilia after 7 days of exposure [19]. Additionally, both i.p. 7-day (FC-and Cas X-treated animals at 25 mg/kg/day) or 30-day (FC at 5 and 10 mg/kg/day) protocols produced diarrhea since the 2nd [19] and 10th dose [47], respectively, leading to weight loss and induced histological alterations suggestive of neurotoxicity, nephrotoxicity, and hepatotoxicity [19,23,47]. So, from a toxicity point of view, the liver and kidneys are the main targets for FC and Casearin X, especially following i.p. exposure, but in subacute treatment conditions, FC causes slight and reversible tissue damage.

On the other hand, non-toxic effects were noted in FC 7-day (25 and 50 mg/kg/day) or 30-day (10 and 20 mg/kg/day)-treated animals by gavage, suggesting it is safer if orally introduced [19], and such findings were confirmed here at 10 and 20 mg/kg/day. Moreover, the seemingly normal hepatic glycogen content detected by PAS staining was a good indication of standard metabolic liver functions [48]. So, FC-induced damages/side effects clearly depend on dosage, route, and time of exposure; none of them were realized in the present investigation.

### 3.2. Analgesic In Vivo Effects

Advances in cancer diagnosis and treatment have improved survival rates for more than five years after diagnosis for about two-thirds of patients, which represents a growing clinical challenge for a large proportion of people with chronic cancer pain. Generally, the management of pain requires the use of analgesics, which has favored the emergence of recurrent side effects [49,50].

Initially, it was used as a non-specific but very sensitive in vivo model of visceral pain (abdominal writhing test) [51] for screening the antinociceptive effects of FC. This classic model induced by acetic acid involves both central and peripheral pathways because it causes hyperalgesia resulting from sensitization of peripheral and spinal cord afferent fibers and the release of prostaglandins, cytokines (IL-1β, IL-6, TNF-α), bradykinin, and sympathetic mediators [52]. Herein, FC acutely or subacutely reduced abdominal writhing by both routes of exposure (25 mg/kg i.p. and 50 mg/kg orally). Historically, *C. sylvestris* roots were prepared by decoction for oral consumption to alleviate different types of pain conditions [53]. Additionally, studies working with aqueous extract (fresh and dry leaf-based teas, 1000 mg/kg), essential oil (125 mg/kg), and crude hydroalcoholic extract (30–300 mg/kg) reported a reduction in acetic acid-induced abdominal writhing [54,55,56].

Since diterpenes are prominent candidates to treat neuropathic and inflammatory pain [57], the paw-licking test induced by formalin was used as a more specific and sensitive protocol to search for analgesic substances. It allows us to distinguish between two phases of nociceptive mechanisms. In this trial, FC reduced paw lickings in all doses and routes tested during the peripheral/neurogenic phase, but only oral doses (25 and 50 mg/kg) showed antinociceptive efficacy in the inflammatory phase. The response in the first (neurogenic) phase is a consequence of the instantaneous release of excitatory amino acids (basically glutamate and aspartate) and an intensively increased activity of primary afferent fibers. On the other hand, later reactions in the second phase are characterized by the release of inflammatory mediators as a result of primary afferent neurons and the release of neuropeptides such as substance P in the spinal cord dorsal region [58,59,60].

Naloxone and L-NAME reduced the antinociceptive effects of FC 50 mg/kg oral, suggesting this pharmacological action of FC is mediated by the peripheral opioid system via µ (mi), δ (delta), and/or κ (kappa) receptors in a similar way to those of morphine and some terpenes [61,62,63,64].

Nitric oxide (NO) plays an important role in acute and chronic pain at both the central and peripheral levels. When formed, it activates the enzyme guanylate cyclase, which increases the cGMP intracellular levels and modulates the activity of many cellular targets, including cyclic guanosine monophosphate (cGMP)-dependent protein kinase as well as ion channels such as ATP-sensitive K^+^ channels. This suggests that agents that facilitate or induce NO synthesis have an antinociceptive effect [65]. It is very likely that nitrergic pathways in pain control demonstrated by FC also have opioid-mediated consequences, inasmuch as morphine acts on peripheral opioid receptors and activates the NO-cGMP pathway, inducing a peripheral analgesic effect, while other studies indicate that NOS inhibition potentiates spinal antinociception in acute and prolonged pain mediated by µ, δ, and, less extensively, k receptors, and also attenuates tolerance for morphine antinociception [66].

In the second, longer-lasting phase, all pharmacological blockers diminished FC antinociception, indicating it is acting as an antinociceptive agent through different but interconnected pathways and ratifying previous studies that show two phases of formalin’s test have different nociceptive pathways thanks to local and/or spinal inflammatory sensitization [58,67].

The FC-reversed action of flumazenil indicates the participation of GABAergic pathways. The periaqueductal gray matter of the midbrain—the most important region for the gate control system—activates descending routes and inhibitory neurons into the lamina II (substantia gelatinosa) of the dorsal horn gray matter by the release of endogenous opioids (enkephalins, dynorphins, and/or β-endorphin), 5-hydroxytryptamine (serotonin), and/or by locus coeruleus-released noradrenaline. Then, the triggering of inhibitory neurons occurs by GABA release. This neurotransmitter blocks neuron-neuron communication through increased gK^+^ and postsynaptic hyperpolarization and/or induces inhibition of glutamate and substance P presynaptic liberation [68].

Some nociception control pathways (e.g., serotonergic and adrenergic ones) are also partially modulated by the cholinergic system. Nicotinic (nicotine) and muscarinic (pilocarpine) agonists display antinociceptive effects in acetic acid-induced contortions and induce the release of inhibitory neurotransmitters (GABA and glycine) [69]. Acetylcholine secretion from central neurons by the activation of presynaptic opioid receptors enhances its analgesic effect [70]. So, naloxone reduces acetylcholine descending release, and systemic administration of morphine can be antagonized by intrathecal injection of muscarinic antagonists, highlighting the link between opioid and cholinergic systems [71]. This connection explains how naloxone reduces the antinociceptive action of FC and denotes the promising analgesic properties of acetylcholine as a neurotransmitter and neuromodulator following release from cholinergic projection neurons and interneurons in the central nervous system [72,73].

Previously, it was shown that higher oral acute doses of hydroalcoholic extract of *C. sylvestris* leaves (100 or 300 mg/kg) were able to increase time for paw withdrawal (hot plate test) and response induced by ovalbumin (hind paw-licking test), but doses as low as 30 mg/kg (close to those used here for FC) were also able to reduce abdominal contortions, whose pharmacological action was annulled by naloxone [56]. Mattos et al. [56] also showed similar outcomes, which clearly suggest that the absence of alterations in the exploratory activity and motor coordination of S180-transplanted animals demonstrates promising antinociceptive properties without behavior alterations.

Interestingly, the pre-treatment with glibenclamide revealed synergistic action. Glibenclamide is both a hypoglycemic sulphonylurea and an ATP-dependent potassium channel inhibitor. As morphine, it works on peripheral opioid receptors and activates NO-cGMP pathways [74]. It is also known that neuropathic or chronic pain similar to one’s resulting from S180 tumor growth or constriction of the sciatic nerve and general nerve endings presents dipyrone–NO-dependent analgesic effects. Prior exposure to sodium nitroprussiate [75] or glibenclamide [65] reduces chronic hyperalgesia caused by sciatic nerve constriction and enhances antinociceptive drug effects in the second formalin test’s phase, which is related to nitric oxide’s role in the inflammatory process, respectively.

Glutamate receptors comprise ionotropic (transmembrane ligand-gated ion channels) and metabotropic receptors, which affect presynaptic glutamate release or indirectly modulate the function of ionotropic receptors. N-methyl-D-aspartate receptors (NMDARs) are heterotetrametric ionotropic receptors that mediate postsynaptic excitatory potentials [76]. It was found that FC could potentially modulate negatively licking time stimulated by glutamate, and a molecular binding was postulated throughout Cas X and NMDA-GluN2 subunits (GluN2A and GluN2B). Indeed, investigations have shown that higher ΔG negative values propose system spontaneity and, consequently, improved stability of the complex, as seen between Cas X and GluN2B [77].

Molecular docking analysis also demonstrated the ability of Cas X to interact with the ligand-binding extracellular domain site of the NMDAR GluN2B subunit, which often affects the conformation of the ion channel between the inactive and active states [78]. Since NMDA receptors with GluN2A/B subunits are the dominant synaptic NMDA receptors expressed within both monosynaptic and polysynaptic input pathways from primary afferent inputs onto lamina I/II neurons [76], the higher affinity of Cas X for GluN2B and related substances with selective targeting for GluN2B would weaken polysynaptic activity following certain types of injury or painful inflammation and would diminish the side effects of a broad-spectrum NMDA receptor medicine.

**Figure 9:**
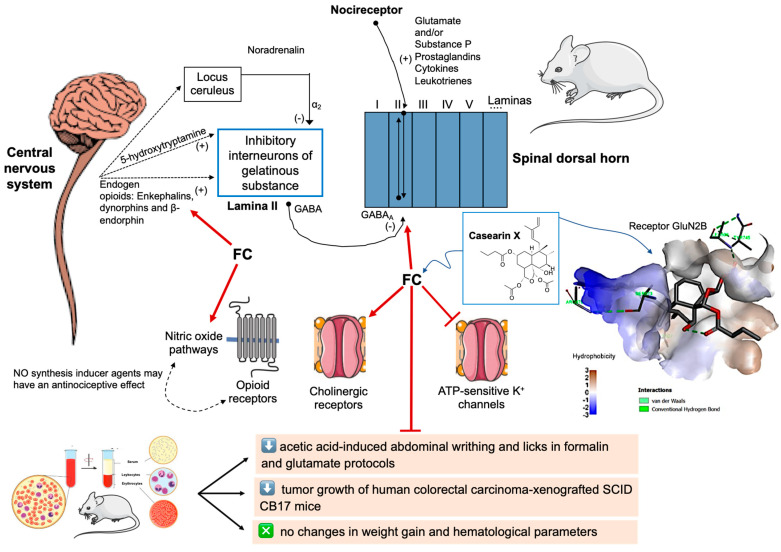
Summary showing how the fraction with casearins (FC) exhibited in vivo antitumor and antinociceptive action and reduced mechanical cancer-related allodynia without side effects. The illustration was partially created in BioRender (free edition).

Because the initial phase of the formalin test is predominantly caused by the activation of unmyelinated C fibers as a result of peripheral stimuli [59], and prior studies indicate central action only at higher doses of *C. sylvestris* leaf extracts (evidenced in the hot plate test), it is supposed that FC has peripheral action, predominantly but likely associated with late inhibition of the release/action of neurochemical mediators in the spinal cord. Then, it is probably the antinociceptive action of FC (when associated with glibenclamide) certainly stimulates downstream excitatory routes (Figure 9), activating lamina II inhibitory interneurons, inducing peripheral analgesic effects by desensitization of secondary afferent neurons, inhibiting glutamate release from presynaptic neurons [79] and/or their action on cognate receptors.

Moreover, FC showed antiallodynic effects by Von Frey analysis in S180-transplanted mice. Since stimulation of the periaqueductal gray matter inhibits reflexes provoked by painful stimuli (e.g., removal of the tail or paw), it is really possible that FC antinociceptive action stimulates downstream excitatory routes, whose descending pathways inhibit the so-called mechanosensitive silent receptors. These receptors play a role when local sensitization occurs from tissue-induced injuries (S180 tumor growth and tissue invasions, for example) and may also participate in hyperalgesia responses [80].

Investigations in vitro and in vivo have already pointed out the anti-inflammatory action of different preparations of *C. sylvestris* (alcoholic/aqueous extracts or essential oils), and they have demonstrated inhibition of edema activity, leukocyte migration, myeloperoxidase activity, nitrogen-derived free radical compounds [22,23,24,26,27,54,55,56], phospholipase A_2_, and tissue proteases [81]. Certainly, the involvement of inflammatory mediators, such as prostaglandins, leukotrienes, cytokines, and biogenic amines, activates and sensibilizes C fiber-related polymodal nociceptors, which play an important role in cutaneous hyperalgesia triggered by mechanical and/or thermal stimuli [15]. This may explain, at least in part, how inhibition of production and/or action of inflammatory mediators are related to the antinociceptive mechanism of FC.

Moreover, the inflammatory process resulting from tissue invasion by S180 tumor cells generates free radicals that supposedly increase glutamate concentrations in the extracellular environment, activating and sensitizing primary afferent neurons, which in turn translates into a higher frequency of nociceptive stimuli [82]. Antioxidant and chemopreventive compounds in *Casearia* leaves [23,24] may block or delay the evolution of inflammatory-related pathological processes, including cancer, reducing acute or chronic-based inflammatory pain, and may suppress mechanical allodynia in murine models of cancer pain [83].

## 4. Materials and Methods

### 4.1. Acquisition of Plant Samples and Phytochemical Analysis

Researchers from the Chemistry Institute of the São Paulo State University collected leaves of *C. sylvestris* at Parque Estadual Carlos Botelho (São Miguel Arcanjo, São Paulo, Brazil/S 24°7′53″, W 47°56′57″) between March and June 2017, and the voucher specimens (AGS04, AGS05, AGS06, AGS13, and AGS19) were registered at the Herbarium Maria Eneida P. Kaufmann (Botanical Institute of São Paulo, Brazil). The fractionation of the ethanolic extract of *C. sylvestris* leaves was performed by solid-phase extraction from silica gel/activated charcoal, followed by normal-phase low-pressure column chromatography over silica gel and preparative reversed-phase (C18) high-performance liquid chromatography. Clerodane/casearin-like diterpenes were identified mainly in the ethyl acetate fraction, named fraction with casearins (FC) by comparison of time of retention (tR) and UV spectra, suggesting the predominance of clerodane diterpenes with λmax between 221–238 nm, mainly [18,30,34], which allowed the purification of Casearin X, but in small quantities. Phytochemical investigations displayed that FC represents an average of 56.5% (mg/g) of the fraction, and Casearin X is the most present compound (around 14.2%) [18,30,34]. All isolated plant samples were kept at −80 °C until dilution and use in pharmacological tests, and HPLC analyses corroborated the phytochemical profile of the fraction. Dimethylsulfoxide (DMSO, Vetec, Recife, Brazil) was used as a co-solvent.

### 4.2. Animals and Ethical Aspects

All procedures for the collection of vegetal samples were registered in SisGen (National System of Management of Genetic Heritage and Associated Traditional Knowledge) (#A00892A and #A33EA7A) and IBAMA/SISBIO (Brazilian Institute for the Environment and Renewable Natural Resources/Biodiversity Authorization and Information System) (#33429-1) and are in accordance with Brazilian Federal Law No. 13,123/2015 about access to national biodiversity [84].

Immunodeficient female CB-17 SCID mice were obtained from the Gonçalo Muniz Institute (Fiocruz, Bahia, Brazil), and male Swiss mice (*Mus musculus*) were obtained from facilities at the Federal University of Piauí (UFPI, Teresina, Piauí, Brazil). All animals were 2 months old with 25–30 g and were housed in sterile cages per group under standard conditions of ventilation, temperature (22 ± 1 °C), and a 12/12 h light/dark cycle, with free access to water and food. Experimental procedures were approved by the local ethics committee for animal use (CEUA/Fiocruz-Bahia #006/2015 and CEUA/UFPI #373/17). They were performed in accordance with the Brazilian law for the use of animals in research (Law number 11.794) and international guidelines on the care and use of experimental animals (Directive 2010/63/EU of the European Parliament and of the Council). All efforts were made to minimize animal pain, suffering, or discomfort. Doses used were based on previous pharmacological investigations of the group [19,21,23].

### 4.3. In Vivo Xenograft Antitumor Model Assay and Systemic-Related Assessments

For this assay, human colon carcinoma HCT-116 cells were maintained in RPMI 1640 medium supplemented with 10% fetal bovine serum, 2 mM glutamine, 100 U/mL penicillin, and 100 μg/mL streptomycin at 37 °C with 5% CO_2_ (Shel Lab CO_2_ Incubator, Baltimore, MD, USA). HCT-116 cells were counted and subcutaneously implanted into the left hind axillary of immunodeficient female CB-17 SCID mice (10 × 10^6^ cells/500 µL/animal) [85]. Mice were randomly divided into seven groups as follows (*n* = 12 animals/group): negative control (DMSO 5% i.p. and DMSO 5% oral by gavage), positive control (5-FU 15 mg/kg i.p.), FC (5 and 10 mg/kg i.p.), and FC (10 and 25 mg/kg oral by gavage). The treatment started one day after inoculation (first day) and was performed for 15 consecutive days.

On the 16th day, the animals were anesthetized with ketamine (90 mg/kg) plus xylazine (4.5 mg/kg) for blood collection from the cardiac artery [86] using sterile tubes and heparinized pipettes to quantify erythrocytes and circulating peripheral leukocytes (XS-1000i-Hematology-Analyzer, Norderstedt, Germany). May–Grünwald–Giemsa-stained blood smears (two per animal) were prepared to confirm the differential amount of white blood cells (WBC) at 400× magnification. The absolute count of a leukocyte subtype was calculated as the product of its respective differential percentage and total leukocyte count [87]. After blood collection, all animals were euthanized with overdosage of sodium pentobarbital (150 mg/kg i.p.), and tumors, liver, kidneys, heart, lungs, and stomach were dissected out, weighed, and fixed in 10% formaldehyde to prepare histological sections (4–7 μm) stained with hematoxylin and eosin (H&E) or periodic acid–Schiff (PAS). Morphological analyses were performed by an expert pathologist under light microscopy (200–400× magnification). The inhibition ratio of tumor growth (%) was calculated according to Ferreira et al. [85].

### 4.4. Antinociceptive Assessments

#### 4.4.1. Acetic Acid-Induced Abdominal Writhing

Mice (*n* = 7 animals/group) received a single dose of DMSO 5% (negative control), 5-FU (25 mg/kg, i.p.), FC (10 or 25 mg/kg, i.p.), oral FC (25 or 50 mg/kg oral gavage), or morphine (5 mg/kg, i.p.) acutely (single dosage) or subacutely (for consecutive 7 days). Then, 30 min after intraperitoneal or 60 min after oral treatment, acetic acid 1% (0.3 mL/animal i.p.) was injected in the peritoneal cavity, or 20 μL of formalin 2.5% was inoculated in the intraplantar region of the right hind paw. After 10 min, the number of contortions (including abdominal muscle contraction and extension of posterior paws) was recorded for 25 min [88].

#### 4.4.2. Formalin Nociception Tests

For the behavioral analysis following formalin injection, animals were placed in a mirrored cage to quantify the licking time in two phases: neurogenic (first phase: 0–5 min) and inflammatory (second phase: 15–30 min) [58,59]. Afterwards, in order to evaluate possible mechanisms involving the antinociceptive response, antagonists of different routes were used: opioid (naloxone, 5 m/kg, i.p.), nitrergic [L-NAME (N-nitro-L-arginine-methyl-ester), 20 mg/kg, i.p.], potassium channels (glibenclamide, 10 mg/kg, i.p.), GABAergic (flumazenil, 2 mg/kg, i.p.), and muscarinic antagonist (atropine, 1 mg/kg, i.p.). Each blocker was administered 30 min prior to the FC (*n* = 7 animals/group).

#### 4.4.3. Glutamate-Induced Nociception

Mice (*n* = 8 animals/group) received a single dose of DMSO 5% (negative control), morphine (5 mg/kg i.p.), or FC (25 mg/kg i.p. or 50 mg/kg oral by gavage). Then, 30 min after intraperitoneal or 60 min after oral treatment, a volume of 20 μL of glutamate solution was injected into the intraplantar region of the right hind paw. Afterwards, each animal was individually placed into a mirrored cage, and the time spent licking paws totaled 15 min. Glutamate injection into the mouse’s hind paw elicits a dose-dependent noxious stimulus characterized by the animal’s behavior of licking the glutamate-injected member [89].

### 4.5. Oncological Pain Model Induced by Sarcoma 180 Tumor

Ten-day-old S180 ascites tumor cells were removed from the peritoneal cavity, counted in a Neubauer chamber by trypan blue exclusion test using an inverted light microscope (Biosystems^TM^, Recife, Brazil) [19], and subcutaneously implanted (10^6^ cells per 25 μL) in the intraplantar region of the right hind paw of healthy Swiss animals [90,91].

Tumor growth was confirmed by changes in paw volumes after 14 days of tumor transplantation. So, all animals were subjected to a baseline evaluation and re-examined again on the 14th day using a paw plethysmometer (Insight, São Paulo, Brazil). The displacement of the liquid (distilled water) is converted into paw tumor volume, comparing the volume of the paw before tumor induction and on the 14th day (Δ, in mL) [92].

On the 14th day, animals were randomly divided into seven groups (*n* = 7 animals/group), and the fraction with casearins dissolved in DMSO 5% was injected at doses (25 mg/kg i.p. and 50 mg/kg/day oral). Negative and positive controls received DMSO 5% and morphine (5 mg/kg, i.p.), respectively.

#### 4.5.1. Open Field Test

Locomotor and exploratory activity were assessed using an acrylic open field with transparent walls and a black floor (30 × 30 × 15 cm) divided into nine equal quadrants. Animals were placed in the center of the apparatus to freely explore it for 5 min. The frequency of line crossings with all four pawns (spontaneous locomotor activity), self-cleaning behavior (grooming), and lifting without touching the walls (rearing) was evaluated. The apparatus was cleaned with a 70% alcohol solution after each behavioral session to remove residue left by the previous animal [93,94].

#### 4.5.2. Rotarod Test

The rotarod test allows the detection of the occurrence of motor incoordination related to a possible muscle-relaxing effect of substances, favoring a more accurate interpretation of nociception tests [95]. After at least one training session on the apparatus, all animals were subjected to the evaluation of movement-induced nociception. Mice were placed with all four paws on a rotating bar (2.5 cm in diameter, 25 cm from the floor, rotating at 17 rpm) for a period of 60 s. The time spent on the rotating bar, in seconds, was recorded.

#### 4.5.3. Mechanical Nociception by Von Frey Assay

Mechanical allodynia was evaluated in the Von Frey apparatus (Insight, Brazil). Each animal was acclimatized for at least 15 min before the behavioral test and individually placed on a transparent acrylic platform (9 × 7 × 11 cm) to allow access to the ventral surface of the hind paws. The frequency of paw withdrawals was obtained through three applications of Von Frey filaments (each stimulus of 1 s each) and with growing intensity (from 0.07 to 10 g). All animals were assessed before tumor inoculation and afterwards at different times [96].

### 4.6. Molecular Docking

The target structures—N-methyl-d-aspartate (NMDA)/glutamate receptors GluN2A and GluN2B—were evaluated using the Protein Data Bank data repository (https://www.wwpdb.org/, accessed on 6 May 2023). All protein structure coordinate archives were obtained from the PDB, in which all polar hydrogen atoms were adjusted to the known protein protonation, and the archives were saved in PDBQT format. Structures with up to 3 Å resolution were obtained. A PDBQT file was also prepared for an early ligand whose conformational energy was minimized in Discovery Studio 2020 but maintained the flexibility of rotational bonds.

The AutoDock Vina software version 4 was used for molecular docking and virtual screening of the binding among Casearin X, the main compound in the FC, and subunits GluN2A/GluN2B of NDMA receptors, producing grid maps. AutoDock Vina-generated files in PDBQT specified the ligand Cas X and receptors. Docking parameters were defined for each complex with a 27,000 Å Grid Box [35]. The parameter value called “exhaustiveness”, the amount of computational effort to obtain a more consistent coupling result, was adjusted to 100 using a command (*./vina--config config.txt--exhaustiveness = 100*). The PyMol software version 2.1.1 was used to find coordination for each complex, to visualize the crystallographic locations of the ligand, and to compare conformation energies. In order to obtain 2D and 3D images of the interactions between ligand and receptor, Discovery Studio 2020 was used.

### 4.7. Statistical Analysis

Outcomes were evaluated by comparing data by one-way analysis of variance (ANOVA) followed by the Newman–Keuls test (GraphPad Prisma, Intuitive Software for Science, San Diego, CA, USA, version 9.1), considering a significance level of *p* < 0.05.

## 5. Conclusions

A fraction with casearins (FC) showed in vivo antitumor action against xenografted human colorectal carcinomas, causing slight conventional chemotherapeutic side effects (Figure 9) and mild and reversible damage in key organs. It exhibited potent antinociceptive activity in acute chemical models, whose results were reversed by opioid, cholinergic, GABAergic, and nitrergic blockers. On the other hand, a sulphonylurea ATP-dependent potassium channel inhibitor showed analgesic synergistic action. The intermolecular energy associated with the different types of interactions throughout Cas X and NMDA receptor-GluN2 subunits (GluN2A and GluN2B) suggests how FC negatively modulates glutamate-induced painful responses. Meanwhile, the mice subchronic intraplantar sarcoma 180 protocol showed FC is efficient for reducing mechanical allodynia without alterations in exploratory and motor parameters. Then, it is supposed that FC has peripheral action, predominantly but likely also associated with inhibition of the release/action of pain neurochemical mediators in the spinal cord. Indeed, FC certainly stimulated downstream excitatory routes, inducing peripheral analgesic effects by desensitizing secondary afferent neurons, inhibiting glutamate release from presynaptic neurons, and/or their action on cognate receptors. These findings support traditional uses of *C. sylvestris* against colorectal carcinomas and emphasize clerodane diterpenes as an emerging class against cancer-related pain conditions.

## Figures and Tables

**Table 1:** Effects of the fraction with casearins (FC) obtained from *Casearia sylvestris* leaves on the wet weight of organs and tumor growth of CB-17 SCID mice transplanted with colon carcinoma (HCT-116) after 15 days of treatment.

Route	Sample	Dose (mg/kg)	Survival	g/100 g of Wet Body Mass					Tumor Mass (g)	% Tumor Inhibition
				Liver	Kidney	Lung	Heart	Stomach		
Intraperitoneal	Vehicle	–	12/12	4.91 ± 0.18	1.52 ± 0.04	0.86 ± 0.03	0.62 ± 0.02	-	1.08 ± 0.07	-
	5-FU	15	10/12	5.16 ± 0.51	1.66 ± 0.08	0.97 ± 0.07	0.68 ± 0.02	-	0.69 ± 0.03 *	36.34 *
	FC	5	12/12	4.71 ± 0.24	1.73 ± 0.04	0.97 ± 0.03	0.67 ± 0.04	-	0.74 ± 0.05 *	31.12 *
		10	12/12	5.41 ± 0.29	1.52 ± 0.07	0.81 ± 0.04	0.61 ± 0.03	-	0.66 ± 0.05 *	39.27 *
Oral	Vehicle	–	12/12	4.81 ± 0.22	1.51 ± 0.06	0.90 ± 0.04	0.60 ± 0.02	1.36 ± 0.13	1.06 ± 0.04	-
	FC	10	12/12	4.80 ± 0.19	1.52 ± 0.05	0.86 ± 0.04	0.59 ± 0.02	1.13 ± 0.08	0.99 ± 0.04	6.05
		25	12/12	4.42 ± 0.16	1.60 ± 0.06	0.99 ± 0.05	0.66 ± 0.05	1.35 ± 0.08	0.70 ± 0.06 *	34.12 *

Values are means ± S.E.M., *n* = 12 animals/group. The negative control was treated with the vehicle used to dilute the drug (DMSO 5%). 5-Fluorouracil (5-FU) was used as a positive control. * *p* < 0.05 compared with the negative control by ANOVA followed by the Newman–Keuls test.

**Table 2:** Profile of the peripheral blood white cells of CB-17 SCID mice bearing HCT-116 colon carcinomas after intraperitoneal or oral treatment for 15 days with a fraction with casearins (FC) extracted from *Casearia sylvestris* leaves.

Route	Sample	Dose (mg/kg)	Erythrocytes (10^6^ Cell/µL)	Total Leukocytes (10^3^ Cél/µL)	Differential Leukocyte Count (%)				
					Basophils	Eosinophils	Neutrophils	Lymphocytes	Monocytes
Intraperitoneal	Vehicle	-	8.23 ± 0.30	3.86 ± 0.34	0	1	73.75	24.25	1
	5-FU	15	7.06 ± 0.91	2.23 ± 0.20 *	0	1	48.00	50.00	1
	FC	5	7.16 ± 0.90	3.71 ± 0.72	0	1	47.60	50.40	1
		10	7.23 ± 0.20	4.40 ± 0.99	0	1	61.00	37.00	1
Oral	Vehicle	-	9.58 ± 0.60	5.06 ± 0.80	0	1	78.89	19.11	1
	FC	10	7.75 ± 0.55	5.05 ± 0.90	0	1	64.00	32.25	1
		25	7.66 ± 0.72	3.14 ± 0.49	0	1	33.80	64.20	1

Values are means ± S.E.M., *n* = 12 animals/group. The negative control was treated with the vehicle used to dilute the drug (DMSO 5%). 5-Fluorouracil (5-FU) was used as a positive control. * *p* < 0.05 compared with the negative control by ANOVA followed by the Newman–Keuls test.

## Data Availability

Data will be made available on reasonable request.
